# Identification and Characterization of Swine Influenza Virus H1N1 Variants Generated in Vaccinated and Nonvaccinated, Challenged Pigs

**DOI:** 10.3390/v13102087

**Published:** 2021-10-16

**Authors:** Álvaro López-Valiñas, Marta Sisteré-Oró, Sergi López-Serrano, Laura Baioni, Ayub Darji, Chiara Chiapponi, Joaquim Segalés, Llilianne Ganges, José I. Núñez

**Affiliations:** 1 Centre de Recerca en Sanitat Animal (CReSA), Institut de Recerca en Tecnologies Agroalimentaries (IRTA), 08193 Barcelona, Spain; alvaro.lopezv@irta.cat (Á.L.-V.); marta.sistere@upf.edu (M.S.-O.); sergi.lopez@irta.cat (S.L.-S.); ayub.darji@irta.cat (A.D.); joaquim.segales@irta.cat (J.S.); llilianne.ganges@irta.cat (L.G.); 2 OIE Collaborating Centre for the Research and Control of Emerging and Re-Emerging Swine Diseases in Europe (IRTA-CReSA), 08193 Barcelona, Spain; 3 OIE Reference Laboratory for Swine Influenza Virus, Istituto Zooprofilattico Sperimentale della Lombardia ed Emilia-Romagna, 25124 Brescia, Italy; laura.baioni@izsler.it (L.B.); chiara.chiapponi@izsler.it (C.C.); 4 Departament de Sanitat i Anatomia Animals, Universitat Autònoma de Barcelona, Bellaterra, 08193 Barcelona, Spain; 5 OIE Reference Laboratory for Classical Swine Fever Virus, IRTA-CReSA, 08193 Barcelona, Spain

**Keywords:** swine influenza virus (SIV), next-generation sequencing, single nucleotide variants (SNVs), nonstructural protein (NS), hemagglutinin (HA), nucleoprotein (NP), neuraminidase (NA)

## Abstract

Influenza viruses represent a continuous threat to both animal and human health. The 2009 H1N1 A influenza pandemic highlighted the importance of a swine host in the adaptation of influenza viruses to humans. Nowadays, one of the most extended strategies used to control swine influenza viruses (SIVs) is the trivalent vaccine application, whose formulation contains the most frequently circulating SIV subtypes H1N1, H1N2, and H3N2. These vaccines do not provide full protection against the virus, allowing its replication, evolution, and adaptation. To better understand the main mechanisms that shape viral evolution, here, the SIV intra-host diversity was analyzed in samples collected from both vaccinated and nonvaccinated animals challenged with the H1N1 influenza A virus. Twenty-eight whole SIV genomes were obtained by next-generation sequencing, and differences in nucleotide variants between groups were established. Substitutions were allocated along all influenza genetic segments, while the most relevant nonsynonymous substitutions were allocated in the NS1 protein on samples collected from vaccinated animals, suggesting that SIV is continuously evolving despite vaccine application. Moreover, new viral variants were found in both vaccinated and nonvaccinated pigs, showing relevant substitutions in the HA, NA, and NP proteins, which may increase viral fitness under field conditions.

## 1. Introduction

Swine influenza is a widely distributed disease that generates important economic losses in the pig industry [1]. The aetiological agents, the swine influenza viruses (SIVs) belong to the *Orthomyxoviridae* family and represent an important threat to public health due to the risk of potential zoonotic infections. The SIV genome is characterized by eight genomic segments of negative-sense, single-stranded RNA, where each segment codes for at least one protein [2]. The proteins of the polymerase complex (formed by two basic polymerases (PB1 and PB2) and one acidic polymerase (PA)), the hemagglutinin (HA) and neuraminidase (NA) surface glycoproteins, and the nucleoprotein (NP) are coded by the RNA segment of the same name [3]. The matrix (M) and nonstructural (NS) genome segment encode each one for two different proteins by splicing mRNA: matrix protein (M1) and ion channel (M2) [4], and nonstructural protein (NS1) and nuclear export protein (NEP) [5], respectively. Moreover, the PB1 segment has an alternative codon frame that codes for the accessory PB1-F2 protein [6].

In the spring of 2009, an outbreak of a new pandemic strain A(H1N1)pdm09 of swine origin was reported in the USA, and it quickly spread to more than 30 countries by human-to-human contact [7]. Since then, according to the Centers for Disease Control and Prevention (CDC), between 151,700 and 575,400 fatal human cases have been recorded [8]. The A(H1N1)pdm09 strain arose from the genomic reassortment of an H1N1 Eurasian “avian-like” (EA) swine virus NA/M and triple reassortants of H1N2 and H3N2 harboring the PB2/PA segments of a North-American avian influenza virus, the PB1 segment from a human H3N2, and the HA/NP/NS segments from a classical swine H1N1 [9].

In a recent published SIV surveillance study, performed on both pigs and farm workers in China, a new emerging genotype 4 (G4) reassortant Eurasian avian-like H1N1 with some pdm09 genes (G4 EA H1N1 virus) was detected. The increasing capability to infect humans and the absence of pre-existing immunity against this strain concerns the possibility of new pandemic virus generation [10].

The SIV evolution, explained under the quasispecies theory, plays an important role in the adaptation, host range, virulence, and emergence of new variants mainly due to both point mutation and genomic reassortment [11]. Although recombination is a mechanism of change, it does not play an important role on influenza virus (IV) evolution [12]. The viral polymerase is characterized by the lack of a proof-reading function during the replication process with a high mutation rate of 10^−3^ to 10^−4^ per gene per generation [13]. Taking that fact into account, the greater accumulation of point mutations on antigenic sites may drive a new antigenic pattern phenomenon known as antigenic drift. Hence, in addition to the high prevalence of IV, antigenic drift may trigger the appearance of new influenza variants able to escape vaccination [14].

Vaccination against SIV is currently the main strategy in order to prevent and control the disease [15]. Nowadays, the most extended vaccines are based on inactivated or attenuated viruses [16] such as the trivalent vaccines that contain the most common circulating SIV subtypes in Europe, EA H1N1, human-like swine H1N2, and human-like reassortant swine H3N2 [17,18]. These vaccines mainly stimulate the IgG production against HA viral proteins and to NA and NP proteins to a lesser extent. These vaccines reduce both clinical signs and viral shedding, although they do not prevent viral replication [19]. With the aim of evaluating the viral evolution and the possible generation of escape mutants, the evolutionary trends of SIV under selection pressure due to vaccination were studied through whole-viral-genome deep sequencing in the present work. Two groups of piglets, vaccinated and nonvaccinated, were infected with the SIV strain of the EA H1N1 subtype. In total, 27 whole viral genome sequences were obtained, and variants detected from both experimental groups were characterized.

## 2. Materials and Methods

### 2.1. Cells and Virus

Madin–Darby Canine Kidney (MDCK, ATCC CCL-34) cells were used for both viral titration and production. Dulbecco’s modified Eagle’s medium DMEM (Lonza, Basel, Switzerland), supplemented with fetal bovine serum (Euroclone, Milan, Italy) (10%), L-glutamine (Gibco Life Technologies, Madrid, Spain) (1%), and penicillin/streptomycin (1%) 100 U/mL (Gibco Life Technologies, Madrid, Spain) was used for the cell culture and cells were further kept at 37 °C with 5% CO_2_ atmosphere.

The A/Swine/Spain/01/2010 H1N1 virus was propagated at a multiplicity of infection (MOI) of 0.01 in the MDCK monolayer cells in the presence of 10 µg/mL of porcine trypsin (Sigma-Aldrich, Madrid, Spain) and then harvested 48 h later. Inoculum titration was performed by serial dilutions in a MDCK cells culture. The 50% tissue culture infection dose (TCID_50_) was calculated following the Reed and Muench method [20].

### 2.2. Experimental Design

Fifteen 5-weeks-old domestic pigs free from SIV and its antibodies were distributed in two groups: group A (piglets from 1 to 8) and group B (piglets from 9 to 15). The numbers of animals used were considered, taking into account similar animal experiments with swine pathogens [21,22,23]. One week after the acclimation period, pigs from group A were vaccinated with the first vaccine dose of an inactivated influenza vaccine (RESPIPORC FLU3, IDT^®^, Dessau-Rosslau, Germany). This vaccine includes the subtypes H1N1 (Haselünne/IDT2617/2003), H3N2 (Bakum/IDT1769/2003), and H1N2 (Bakum/1832/2000). According to the manufacturer’s instructions, 2 mL of vaccine was intramuscularly administered per pig. After 21 days post-vaccination (dpv), a second immunization was carried out. In parallel, group B pigs were intramuscularly inoculated with the same volume of PBS (nonvaccinated group).

Three weeks after the second immunization (42 dpv), animals from both groups were challenged using two administration routes: intranasal (1 mL per nostril) with a diffuser device (MAD Nasal, Teleflex, Morrisville, NC, USA) and endotracheal (5 mL), by intubation of the animal restrained with an immobilizing pig lasso, both with 10^6^ TCID_50_ of the A/Swine/Spain/01/2010 (H1N1) [24]. After challenge, pigs were monitored daily for clinical signs and animal behavior in a blind manner by trained veterinarians. Registered clinical signs were scored from 0 to 3 as previously described [25]. After challenge, animals were euthanized at 2, 5, and 10 days post-infection (dpi): 3 animals per group at 2 dpi, another 3 per group at 5 dpi, and the remaining piglets at 10 dpi. Animal experiments were performed in AM Animalia facilities (La Vall de Bianya, Girona, Spain). All procedures were approved by the animal ethics committee from the *Generalitat de Catalunya*, under the project number 9657 (24.01.2018), preserving the Spanish and European regulations.

### 2.3. Sample Collection

Nasal swab samples were collected before the first and the second vaccination, at the viral challenge day and at 1, 2, 3, 5, 7, and 10 dpi. Blood samples were collected before each vaccination, at challenge and before necropsy.

Moreover, lung and nasal turbinate (NT) tissues were taken at necropsy and were homogenized in brain heart infusion medium (10% weight/volume). Broncho alveolar lavage fluid (BALF) was collected by filling the right lung of each piglet with 150 mL of PBS, and after lung massage liquid was collected [26]. An extra lung tissue sample was also collected and stored in formalin.

### 2.4. Detection of Antibodies against Influenza Nucleoprotein

The presence of antibodies against influenza np was assessed using the ID Screen^®^—influenza A Antibody Competition ELISA kit (ID VET, Grabels, France). The ELISA and the inhibition percentage value calculations were performed following the manufacturer’s instructions. Inhibition percentage values below 45% were considered positive, whereas values higher than 50% were negative. Values between 45 and 50% were considered doubtful.

### 2.5. Pathological Analyses in Lung and Immunohistochemistry to Detect SwIV

Animal necropsies were performed, including a macroscopic examination of the lung parenchyma. The percentage of the lung-affected area was calculated by image analysis (IA), as previously described [27], using the software ImageJ^®^ (https://imagej.nih.gov/ij/ (accessed on 10 September 2020)). The *T*-test was applied to study statistical differences between both groups.

The lung from each individual was fixed by immersion in 10% buffered formalin, dehydrated, and embedded in paraffin wax. For the examination under light microscopy, paraffin blocks were sectioned at 3–5 μm cuts and stained with hematoxylin-eosin (HE) [28,29].

Influenza virus detection by immunohistochemistry (IHC) in the lungs was based on a two-step polymer method (Envision TM) [30]. For this purpose, monoclonal antibodies against influenza A virus (IgG2a, Hb65, and ATCC) and system + HRP-labeled polymer Anti-Mouse (K4001, Dako) were used as primary and secondary antibodies, respectively. To microscopically score lung lesions, a semiquantitative method based on affected airways and amount of immunoreactivity were used, respectively [31].

### 2.6. Viral RNA Detection

BALF, nasal swab, lung, and NT supernatants collected throughout the study were further analyzed to isolate, detect, and quantify SIV. Therefore, viral RNA was extracted from samples using MagAttract 96 Cador Pathogen kit ^®^ (Qiagen, Düsseldorf, Germany) according to the manufacturer’s instructions. To quantify the viral RNA of each sample, the quantitative reverse transcription-PCR (RT-qPCR) assay based on the amplification of the conserved segment of the matrix (M) gene was performed in the Fast7500 equipment (Applied Biosystem). The amplification reaction conditions were: 0.4 µM of forward primer (M+25), 0.4 µM of reverse primer (M-124), 0.3 µM of probe (M+64 FAM-TAMRA), 3 µL of extracted RNA, and 0.8 µL of one-step RT-PCR Master Mix Reagents (Applied Biosystems, Foster City, CA, USA) [32]. Threshold cycle (Ct) values equal to or lower than 40 were considered positive. Samples in which fluorescence was undetectable were considered negative.

### 2.7. Whole Influenza Genome Sequence by NGS

The A/Swine/Spain/01/2010 (H1N1) inoculum and samples with an RT-qPCR Ct value lower than 35 were used for whole-genome sequencing, by simultaneous amplification of the eight influenza RNA segments [33]. In addition, to enhance the amplification of the longest segments (PB1, PB2, and PA), a second amplification reaction was performed, according to a previously described protocol [34]. The amplification reaction conditions were: 0.2 μM of forward primer (MBTuni-12 or MBTuni12G), 0.2 μM of reverse primer (MBTuni-13), 2.5 μL of extracted RNA, and 0.5 μL of SuperScript^®^ III One-Step RT-PCR System with Platinum™ Taq High Fidelity DNA Polymerase (Thermo Fisher Scientific, Waltham, MA, USA).

Samples with the eight influenza segments amplified [33] were sequenced by Illumina technology. Multiplexed sequencing libraries were created following the Nextera-XT DNA Library Prep protocol (Illumina^®^, San Diego, CA, USA). Libraries were sequenced using a Miseq Reagment Kit v2 in a 150 cycle paired-end run, on a Miseq Instrument (Illumina^®^, San Diego, CA, USA). Finally, Illumina adapters were automatically removed from FASTQ files. Sequencing data were deposited at the National Center for Biotechnology Information (NCBI, https://www.ncbi.nlm.nih.gov/ (accessed on 14 September 2021)), with the accession number (PRJNA763061).

### 2.8. Mapping and Variant Calling

The reads quality of raw data was verified by FastQC (v 0.11.8) [35]. Low-quality reads (Phread < 30) were removed with Trimmomatic (v0.39) [36]. Subsequently, the read alignment tool Bowtie2 (v2.3.5) [37], using the *very-sensitive* function, was used to align the A/Swine/Spain/01/2010 (H1N1) inoculum reads against the reference SIV genome sequences (JX908038-45) [38]. Post-mapping, unmapped reads, and reads with a quality map lower than 30 were removed with Samtools (v.0.39) [39]. Moreover, PCR duplicates were removed with the Picard “MArkDuplicatesSpark” command option and reads were recalibrated with “BaseRecalibrator,” both included in GATK4 (v4.1). Finally, a consensus sequence was generated with Bcftools (v.1.9) using the *consensus* option [39].

To study possible single nucleotide polymorphisms, all sequenced samples were mapped against the previously generated consensus sequence. Subsequently, the mapped read count per position and per genomic segment of each sample was calculated with the “-*depth*” function included on Samtools (v1.9) and plotted with the ggplot2 library [40] using RStudio software [41].

The variant calling file (VCF) of each sample was generated with LoFreq software (v2.1.5) using default parameters [42]. To define a single nucleotide variant (SNV), the following requirements were considered: mapping quality > 30, minimum coverage depth of 50, alternative base supported by at least 10 reads, and *p* value per change found <0.01. The effect on variants was predicted by SnpEff software (v.4.3) [43], where the H1N1 SIV database was previously built with the “build–gtf22” function. Finally, SNVs with an allele frequency lower than 1% were excluded from the analysis. Moreover, a chi-squared test was performed to study statistical differences between the proportion of synonymous and nonsynonymous SNVs with allele frequencies greater than 1 and 5% in both experimental groups. *p* values < 0.05 were considered significant.

### 2.9. Quasispecies Analysis

The nucleotide diversity (π) of each sample population, including inoculum, was calculated with SNPGenie software as follows [44,45]:

$$\pi =\underset{i<j}{\sum }\frac{d_{ij}}{\left(n^{2}-n\right)/2}$$

where π of each segment is given by the mean number of changes per site (*d_ij_*) divided by all pairs of sequences at that site; thus, n is the coverage depth on site. In addition, analysis of variance (ANOVA) and the subsequent Kruskal–Wallis post hoc test by rank were performed to study statistical π differences between groups, segments, and collected samples per day. All these analyses were performed using RStudio [41]. Kruskal–Wallis *p* values < 0.05 were considered significant. Samples with a mean depth per segment lower than 100 were excluded from this analysis.

## 3. Results

### 3.1. Kinetics of RNA Viral Detection and Antibody Induction after Vaccination and SIV Challenge

**Figure 1:**
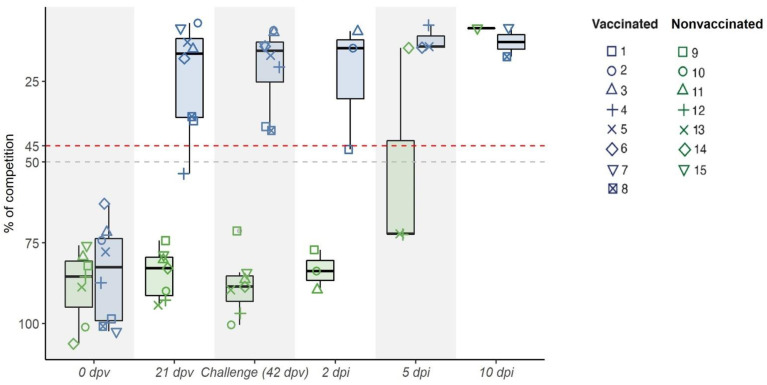
Kinetics of the IgG antibody levels against the NP detected by ELISA in sera after vaccination and viral challenge. IgG antibodies measured as percentage of competition at first vaccination (0 dpv), 21, 42 dpv (day of challenge) and at 2, 5, and 10 dpi are depicted. Points above the dashed red line (competition > 45%) are considered positive, whereas points under the dashed grey line (competition < 50%) are considered negative. Values between the two lines are considered doubtful. Groups of vaccinated and nonvaccinated animals are shown in blue and green, respectively.

After the first vaccination, an antibody response to the NP protein was found in seven out of eight vaccinated animals (group A), all being positive three weeks after the second immunization (day of viral challenge) (Figure 1). However, at viral challenge, lower antibody levels near to the cut-off were found in two vaccinated pigs (number 1 and 8). After challenge, similar values were maintained by most immunized pigs until the euthanasia day, except for pigs 1 and 8, which increased over time. Meanwhile, nonvaccinated pigs remained negative during the trial, except for pigs 14 and 15, which resulted to be positive at 5 and 10 dpi, respectively (Figure 1).

After SIV challenge, higher RNA viral loads were detected in the majority of nasal swab samples collected from nonvaccinated pigs as early as 1 and until 5 dpi. By contrast, lower RNA loads were detected in pigs from the vaccinated group (Table 1). Nonetheless, RNA viral shedding was found from day 2 until day 5 in all vaccinated-challenged pigs. Throughout the days after challenge, a decrease in viral load was observed, until no SIV RNA detection at 10 dpi. Likewise, in the case of pulmonary tissue and BALF, higher RNA loads were found in all nonvaccinated pigs from 2 to 5 dpi. Although lower RNA loads were also found in the lung and BALF from vaccinated-challenge pigs, the RNA levels were higher than those detected in nasal swabs (Table 1). The viral load was higher in the lung at 2 dpi with values between 26 and 21, in animals from both groups. In NT, only three samples were positive, two from 2 dpi and one from 5 dpi (Table 1).

### 3.2. Lung Lesions Determined in Vaccinated and Nonvaccinated Challenge Pigs

Gross lung lesions were detected in both vaccinated (13.41 ± 9.38 of affected lung) and nonvaccinated (33.03±15.7 of affected lung) pigs (Table 2), with these differences being significant (*t*-test; *p* = 0.0105). Similar microscopic lesion scorings were found in both experimental groups (Table 2), with the highest scores being registered at 2 and 5 dpi. Remarkably, the viral labeling detected by immunohistochemistry in lung sections was higher in vaccinated animals at 2 dpi. At 5 dpi, low viral immunodetection was found in nonvaccinated animals, whereas vaccinated ones remained negative.

### 3.3. Determination of Whole-Genome Sequences from Vaccinated and Nonvaccinated Animals after A/Swine/Spain/01/2010 (H1N1) Challenge

**Figure 2:**
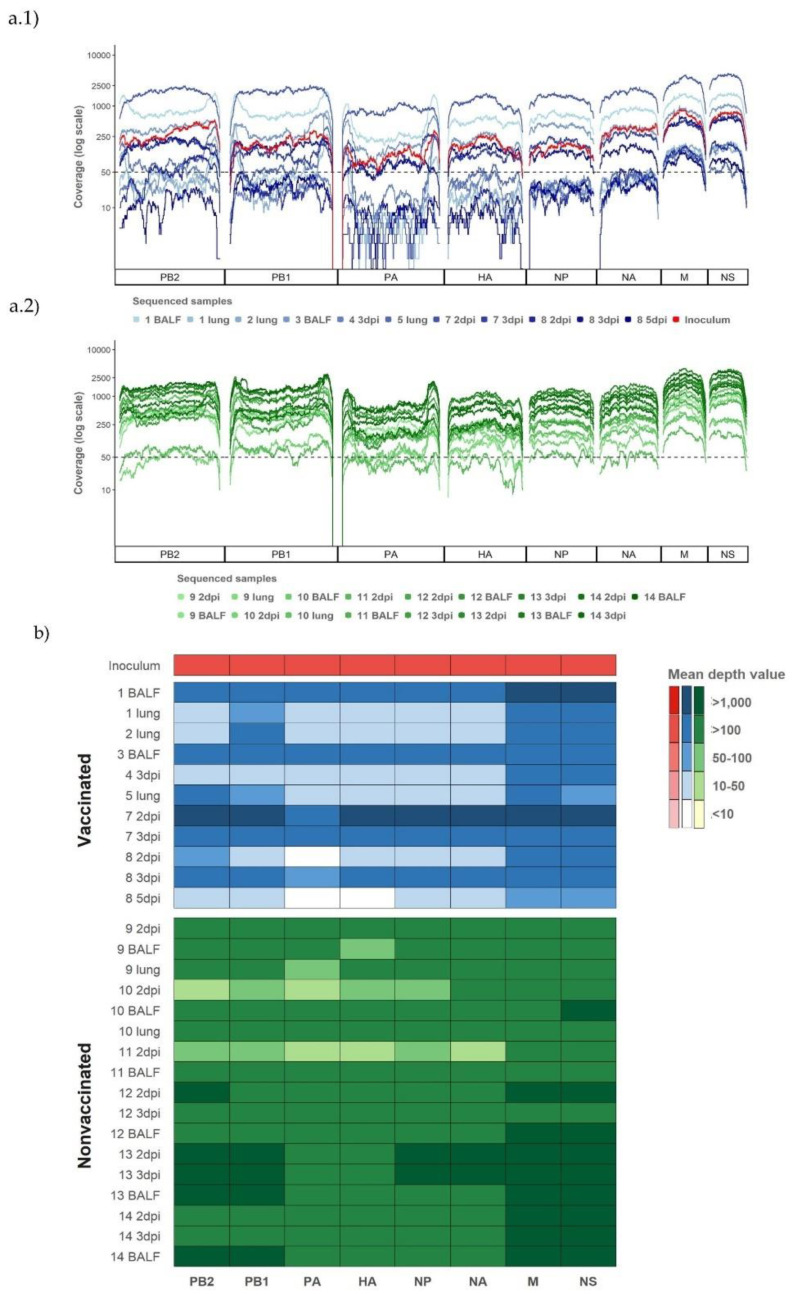
Coverage per genomic segment and sample from both experimental groups and the challenge inoculum. (**a**) Representation of the coverage of Illumina sequencing reads mapped against A/Swine/Spain/01/2010(H1N1) used for challenge. (**a1**) Sequencing profiles of sequenced samples from vaccinated animals and inoculum plotted in different tones of blue and red, respectively. (**a2**) Sequencing profile of sequenced samples from nonvaccinated animals plotted in different tones of green. (**b**) Representation of the mean read coverage heat map per segment. The identification of the animal from which the samples come and the kind of collected sample are specified on the figures, with the nasal swab collected at the indicated day being the samples with dpi.

From a total of 37 samples, 28 influenza genomes were amplified and sequenced, 11 from vaccinated animals and 17 from nonvaccinated ones (Table 1 and Figure 2). In addition, the complete genome sequence from the A/Swine/Spain/01/2010 (H1N1) strain used as inoculum was also obtained.

After quality control, as an average, all processed reads had a length of 126.79 (±4.3) nucleotides and 64,966 reads per sample, reaching a total of 1,948,362 reads for all samples. After mapping and duplicates removal, we obtained samples between 201,577 and 2227 reads, both from nasal swab samples collected in vaccinated pigs. The highest number of reads corresponded to animal 7 at 2 dpi and the lowest to pig 8 at 5 dpi. This last sample was removed for further analysis. Finally, we obtained 1,620,216 reads that were included in the genetic diversity analysis (Table S1).

The majority of SIV genomic segments were sequenced with a coverage greater than 50 reads per position in all samples, and 309,214 out of 379,204 (81.54%) positions sequenced were represented with more than 50 reads, reaching 4378 reads as a maximum. In general, the short segments (NS, M, NA, and NP) had a better read depth per position than the larger ones did, with the segment of polymerase PA being the segment with the lowest depth (Figure 2). In total, all the eight genome segments sequenced, with a mean depth value greater than 100, were obtained for 5 and 15 samples from the vaccinated and nonvaccinated animals, respectively. By contrast, low coverage in all segments, except in NS and M, was found in the eight remaining samples from pigs 1, 2, and 5 in the lung, from pigs 8, 10, and 11 in nasal swabs at 2 dpi, and from pig 4 at 3 dpi also in the nasal swab (Figure 2a,b).

### 3.4. Genetic Variation between SIV Samples Collected from Vaccinated and Nonvaccinated Pigs

**Figure 3:**
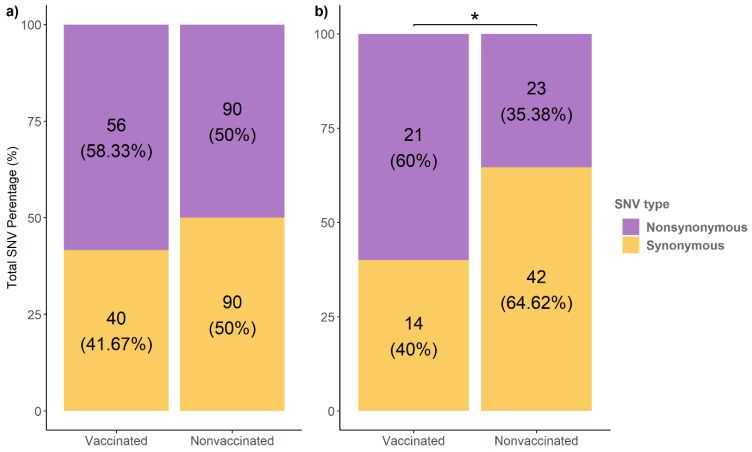
Total number of synonymous and nonsynonymous SNVs found from vaccinated and nonvaccinated animals. (**a**) Substitutions with an allele frequency greater than 1%. (**b**) Substitutions with an allele frequency greater than 5%. * *p* < 0.05.

The analysis of the mapped reads was performed to study the viral population dynamics in both vaccinated and nonvaccinated animals after viral challenge. A total of 276 SNVs with an allele frequency greater than 1% were identified from viral populations recovered from both experimental groups (Figure 3). Notably, in vaccinated animals, 40 synonymous and 56 nonsynonymous variants were identified, representing 41.6 and 58.33% of the total number of SNVs found in this group, respectively. By contrast, the same number of synonymous and nonsynonymous variants (90 SNVs) were registered in the nonvaccinated group (Figure 3a).

In addition, 100 variants with an allele frequency higher than 5% were identified. The 60% of variants found in vaccinated pigs were nonsynonymous, whereas this percentage decreased to 35.38% in nonvaccinated animals, with this difference in frequencies being significant (chi-squared; *p* = 0.03123). Therefore, a total of 44 nonsynonymous substitutions with an allele frequency greater than 5% were detected (Figure 3b).

**Figure 4:**
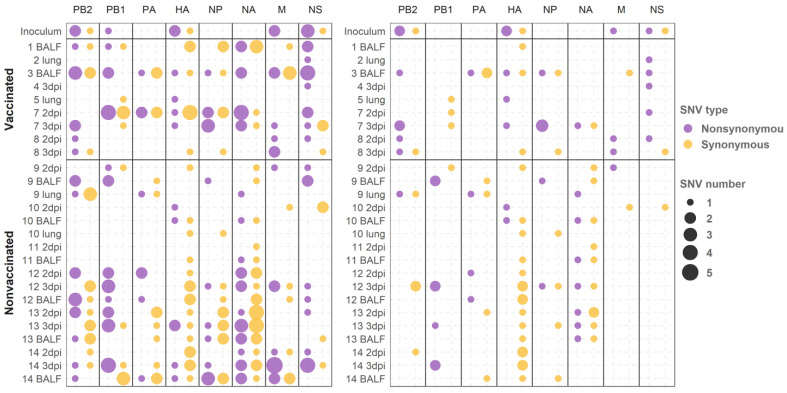
Genome segment distribution and number of synonymous and nonsynonymous SNVs found from sequenced samples from vaccinated and nonvaccinated animals. (**left**) Substitutions with an allele frequency greater than 1%. (**right**) Substitutions with an allele frequency greater than 5%.

In general, amino acid substitutions were identified in all segments along the genome in both experimental groups (Figure 4). In vaccinated animals, 21 amino acid substitutions with an allele frequency greater than 5% were identified in PB2 (M567I, T98K, S506P, R310K, and G586R), PA (Y130C), HA (V521M, V233I, and I513V), NP (D289E, A122E, A232T, and G281V), NA (S354N), M1 (T113A), M2 (S73N), and NS1 (R81S, E179A, E65G, G161E, and D67N); by contrast, 23 were found in nonvaccinated animals: PB2 (E31G, P67L, and H445Q), PB1 (N328S, I322V, M195I, G101R, and T156I), PB1-F2 (E31G and P67L), PA (F646S, G81R, and V618I), HA (I278V and D200N), NP (K97R and E243G), NA (V379I and S354N), and in M1 (T48N). For further variant details and variants noted with an allele frequency greater than 1%, see the supplementary data (Table S2).

### 3.5. Nucleotide Diversity in the Viral Population at Different Time Points

**Figure 5:**
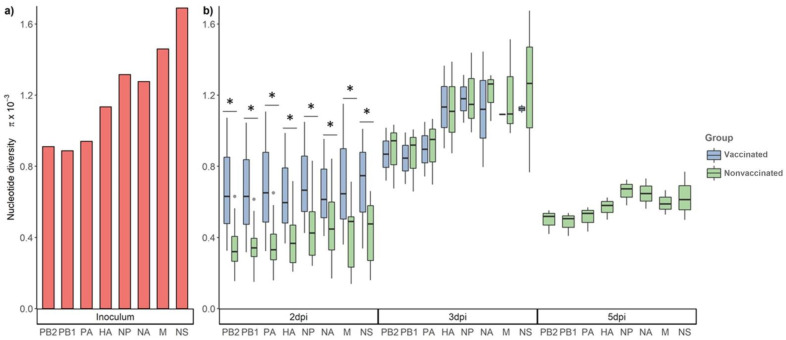
Nucleotide diversity (π) in the viral population at different time points. (**a**) Nucleotide diversity bars of inoculum sequence. (**b**) Box plot of viral population nucleotide diversity from all collected samples at 2, 3, and 5 dpi from vaccinated and nonvaccinated groups. Whiskers represent variability outside the lower and upper quartiles of each represented box. * *p* < 0.05.

For the study of the nucleotide diversity on each genetic influenza segment, those samples with at least one segment with a mean depth value lower than 100 reads were excluded for the analysis (Figure 2b). The nucleotide diversity was higher in the inoculum, then it decreased in samples collected at 2 dpi in both experimental groups (Figure 5). In addition, the π was significantly higher at 2 dpi in vaccinated animals in comparison with that of the nonvaccinated group. However, at 3 dpi, the nucleotide diversity was higher in both groups in comparison with the previous day, and significant differences were not found in any segment from the viruses recovered from both studied groups. In addition, at 5 dpi, the π value decreased in nonvaccinated animals, while no sequences were determined in samples from vaccinated animals (Figure 5).

## 4. Discussion

In recent decades, the increase in pork meat production has caused significant global growth in the swine industry that contributes to the spread and maintenance of swine pathogens [46]. Swine influenza viruses are highly genetically diverse and distributed worldwide in pigs. These pathogens cause significant losses to the porcine industry and pose a continuous threat to both human and animal health due to the difficulty of formulating a universal effective vaccine to control disease [47]. Nowadays, the most extended vaccination strategies used against SIV are based on attenuated and inactivated vaccines that do not provide full protection against infection [19]. Vaccination can modify viral evolution trends, as the natural selection process could boost the accumulation of point mutations on antigenic sites, allowing the virus to escape to preexisting immunity, as previously reported [48,49]. Knowing the biological mechanisms that shape SIV genetic diversity is crucial to understand the evolution of the virus and its implication in host range, antigenicity, virulence, antiviral resistance, and pathogenesis [11]. Therefore, in the present study, differences in evolution trends and genetic diversity between viruses collected from those previously vaccinated with a trivalent vaccine and nonvaccinated animals were studied after an inoculation of a SIV H1N1 subtype by NGS.

In the present work, viral replication and pathological findings were observed in both vaccinated and nonvaccinated pigs, as already reported [50]. However, viral shedding was lower and pathological findings were milder in vaccinated animals. Therefore, the vaccine used in the present study demonstrated its efficacy by means of less severe lesions and lower viral loads in vaccinated animals. This fact had a direct impact on sequencing results, as more samples could be sequenced from nonvaccinated animals and with greater coverage per genomic sample in comparison with samples obtained from vaccinated animals. Nevertheless, full SIV genome sequences from vaccinated animals could also be obtained, and SNVs were found in all sequenced samples from both experimental groups, evidencing intra-host variability.

Considering the total number of SNVs found with an allele frequency greater than 1%, the same proportion of synonymous and nonsynonymous variants were detected in samples from nonvaccinated animals, indicating that, in this scenario, neutral selection pressure may be involved in driving the SIV evolution. When considering variants with an allele frequency greater than 5%, the nonsynonymous variant proportion was greater than synonymous ones. This finding may indicate a purifying selection event where the deleterious mutations are being eliminated, as previously reported. [51,52]. Thus, no positive selection pressure was found in nonvaccinated animals. Meanwhile, in vaccinated animals, the proportion of nonsynonymous variants found was greater than the synonymous ones, analyzed by both 1 and 5% of allele frequency. This analysis may support the viral evolution under positive selection pressure in vaccinated animals [53,54,55]. Based on obtained results, SIV could increase its fitness in both vaccinated and nonvaccinated animals, favoring viral maintenance in the field. Similar findings had been described for classical swine fever virus affecting pigs [56].

Synonymous and nonsynonymous variants were detected along all segments in sequenced samples from both experimental groups (Figure 4). Considering variants with an allele frequency greater than 5%, which could have a greater impact on virus fitness, nonsynonymous substitutions were mainly found in the PB2, HA, NP, and NS1 SIV proteins in samples collected from vaccinated animals. On the contrary, in samples from nonvaccinated animals, most viral changes were found in PB1.

**Figure 6:**
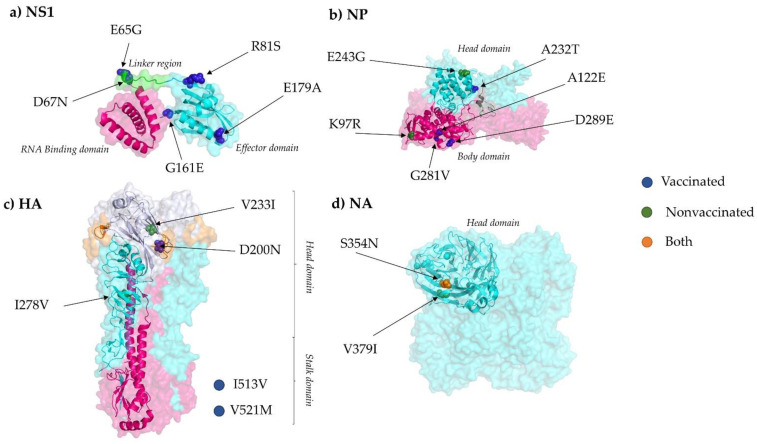
Location of all substitutions (allele frequency > 5%) described in this study in NS1, NP, HA, and NA proteins. (**a**) NS1 protein (PDB accession no. 4OPH) [61] RNA Binding (RBD), Linker region, and effector domain (ED) are highlighted in pink, green, and cyan, respectively. (**b**) NP trimer protein (PDB accession no. 2IQH) head domain is indicated in cyan, whereas body domain is highlighted in pink [64]. (**c**) HA trimer is also represented (PDB accession no. 3LZG); cyan, pink, and blue domains represent HA1, HA2, and Receptor binding domains, respectively. In orange, the CA antigenic site is highlighted [65]. I513V and V521M substitutions are not in the limit of the crystallographed structure. (**d**) NA tetramer (PDB accession no. 4B7Q) protein [66]. Finally, highlighted substitutions in blue were found in vaccinated animals, those in green were found in nonvaccinated animals, and those in orange in both. The PyMOL Molecular Graphics System, Version 4.3 was used to visualize the protein structures.

Five nonsynonymous substitutions were detected within the viral NS1 protein recovered only in samples from vaccinated animals (Figure 6a). The role of NS1 protein in the translation of viral mRNAs and interferon regulation has been previously described. This protein has been associated with the regulation of viral replication and host immunity, including the interferon mechanism regulation [57,58,59,60]. NS1 has two structural domains linked by a flexible linker region (LR): the RNA binding domain (RBD) formed by the 73 first residues and the effector domain (ED) from residue 85 until the end of the molecule [61,62,63] (Figure 6a). Considering that, the substitution E65G located in the RBD, D67N in the LR, and R81S, G161E, and E179A located in the ED from the NS1 protein may regulate the SIV replication capacity, the pathogenicity, and the immune response.

Substitutions E65G and D67N could play roles in the plasticity of the molecule, resulting in a different conformation of the NS1 protein and allowing it to bind to different host substrates, affecting its activity [67]. Likewise, the R81S substitution is allocated in a domain that interacts with the host eukaryotic translation initiation factor 4G1 (eIF4G1). The recruitment of this factor by NS1 promotes viral mRNA translation instead of host mRNA [68,69]. A previous study demonstrated the relation between this region and pathogenesis in birds with H5N1 [70]. This substitution was detected with an allele frequency of 18% in a viral sample collected in the lung of animal 2, which had the maximum histopathological score. Therefore, the variation in NS1 protein could have a contribution to both mRNA viral translation and pathogenesis. Another substitution found inside the NS1 protein was the G161E. As previously reported, this position was allocated in the human leukocyte antigen B7 (HLA-B7) [71] binding motif that could be equivalent to the swine leukocyte antigen B7 (SLA-B7). Thus, this substitution may affect the antigen presentation capability by the host. The last substitution found in the NS1 was the E179A. Notably, a mutation in this position was previously described in the host cleavage and polyadenylation specificity factor (CPSF) binding domain [72]. The interaction with CPSF subunit avoids the correct host pre-mRNAs processing, inhibiting the 3′end cleavage and the polyadenylation [73]. Substitutions in the same position had also been found in the equine and canine influenza virus H3N8 subtype. A mutation in this position may have a great impact on the adaptation ability of avian influenza viruses to the horses [74]. In addition, this substitution on the canine influenza virus is related to the ability of the virus to inhibit the host gene expression [72]. Interestingly, this substitution was found in a viral sample recovered from animal 3, which developed a severe lung lesion despite being vaccinated. Therefore, this substitution may play a role in disease pathogenesis. The changes reported in the present study support the high relevance of one or a few substitutions in the NS1 protein on viral replication and host adaptation, as previously reported [74,75,76,77].

In the case of the NP, it has been associated with the RNA synthesis and trafficking, and also interacts with viral polymerases and host polypeptides [78,79]. Three substitutions were simultaneously noted in a viral sample sequenced from vaccinated animal number 7 at 3 dpi: A122E and G281V in polymerase interaction sites of the body domain and A232T in the head domain [65,80] (Figure 6b). In addition, substitution D289E in the body domain of the protein was also detected in vaccinated animal 3. On the other hand, two substitutions were allocated in the head and body domains, E243G and K97R, respectively (Figure 6b). It should be highlighted that in the inoculum consensus sequence, other arginine residues were allocated in R292, R421, and R445. Four arginines inside NP were previously described as interferon (IFN)-inducible tripartite motif 22 (TRIM22) protein resistance [81]. This resistance avoided the interaction between NP and TRIM22, preventing viral polyubiquitination and subsequent degradation by proteasome [82]. This arginine signature was reported in A(H1N1)pdm09; however, the lysine (K) signature at the four residues was being kept in seasonal influenza until 2009. Besides, the arginine signature was also described in the IV derived from the 1918 pandemic flu [81]. Therefore, fixation of the K97R substitution in nature, found in nonvaccinated animal 9, could pose a risk for both animal and human health due to its similarity to previous flu pandemics.

The HA protein has been associated with the binding to the host acid sialic receptor, causing membrane fusion within the endosome, allowing virus cell entry [83]. This protein is an important target to generate neutralizing antibodies during IV infection [84]. Notably, in this study, five substitutions were found in the HA protein, three in vaccinated animals and two in nonvaccinated animals (Figure 6c,d). Two substitutions were found in the receptor binding domain (RBD) of the HA protein, the D200N and V233I, both allocated close to the Ca antigenic site according to the antigenic structure of CA04 HA from the 2009 H1N1 pandemic virus [65,85]. Substitutions in the RBD were also found in vaccinated and nonvaccinated pigs in a previous study [86] (highlighting the relevance of this region that could affect the host range. The substitution V233I was recovered with high allele frequency (30.4%) from a vaccinated animal (number 5) that, after viral challenge, showed moderate clinical signs and severe lung lesions. This substitution may be involved in evading the pre-existing immunity in this animal after vaccination. The other two substitutions, I513V and V525, both from vaccinated animal samples, are allocated in the transmembrane region of the stalk domain of the protein. Substitutions in this domain could change its interaction with surrounding lipids and its structural properties, changing the antigenic exposure of the HA, allowing the virus to avoid the immune response [87]. Finally, the I278V substitution was allocated in the esterase subdomain [85].

The NA protein plays an important role at the final stage of IV infection by interaction with host sialic acid, releasing the virion progeny [88]. This protein has also been described as one of the main targets to generate neutralizing antibodies during IV infection [84]. Two nonsynonymous substitutions were found in the head domain of the NA, the S354N, located in the second sialic acid binding site [89] and the V379I (Figure 6d). The substitution S354N originated from a new N-glycosylation site (NSSS), fulfilling the requesting motif N-X-S/T-X, where X is any amino acid except proline. Changes in N-glycosylation sites can affect properties of viral surface antigens and virions by acting in viral incorporation and replication [90]. This substitution was found in both experimental groups; this could indicate two different scenarios: a selective advantage independent of the vaccination or virus readaptation from MDCK cells to the porcine host.

Our findings suggest, once again, the broad range of adaptation and evolution capacity of SIV. Herein, the impact of each substitution found is hypothesized according to previous literature. Further analysis, including viral transmission assays, should be performed to study deeply the impact of each substitution on viral fitness. Overall, the increasing pig population in Europe and the continuous persistence of SIV in farms allow SIV to continuously evolve, affecting its host range, antigenicity, virulence, antiviral resistance, and pathogenesis. Therefore, the generation of a new pandemic SIV is feasible, as with what happened in the 2009 H1N1 IV pandemic. Stricter vaccination schedules should be carried out on farms to avoid maximum SIV circulation, increasing the current percentage of the swine population vaccinated against SIV in Europe (10–20%) [91].

## Figures and Tables

**Table 1:** RT-qPCR Ct value results obtained from viral samples extracted from NS at different time points and from the lung, BALF, and NT.

Group	Pig ID	Nasal Swab						Euthanized Day		Tissue Samples	
		1 dpi	2 dpi	3 dpi	5 dpi	7 dpi	10 dpi		BALF	LUNG	NT
*Vaccinated Group*	*1*	35.8	38.7	†	†	†	†	*2 dpi*	30.2	25.7 *	35.2
	*2*	*Neg.*	39.4	†	†	†	†		**30.4**	23.3 *	*Neg.*
	*3*	*Neg.*	37	†	†	†	†		33,9	*Neg.*	*Neg.*
	*4*	*Neg.*	*Neg.*	34.14 *	*Neg.*	†	†	*5 dpi*	36.7	35.1	*Neg.*
	*5*	*Neg.*	*Neg.*	**33.8**	*Neg.*	†	†		35.4	32.31 *	*Neg.*
	*6*	*Neg.*	*Neg.*	37	36.5	†	†		*Neg.*	*Neg.*	*Neg.*
	*7*	38.4	30.7	34	*Neg.*	*Neg.*	*Neg.*	*10 dpi*	*Neg.*	*Neg.*	*Neg.*
	*8*	*Neg.*	35.72 *	33.2	34.11 *	*Neg.*	*Neg.*		*Neg.*	*Neg.*	*Neg.*
*Nonvaccinated Group*	*9*	34.8	31.3	†	†	†	†	*2 dpi*	28.4	21.4	28.8
	*10*	36.1	34.79 *	†	†	†	†		22.8	24.9	*Neg.*
	*11*	*Neg.*	33.42 *	†	†	†	†		29	**21.8**	*Neg.*
	*12*	30.1	28.9	32.8	**33.8**	†	†	*5 dpi*	29.8	**26.3**	39.2
	*13*	26.9	28	29.3	*Neg.*	†	†		29.7	**30.4**	*Neg.*
	*14*	32.9	29.9	32.8	*Neg.*	†	†		28.6	**30.2**	*Neg.*
	*15*	39.67	36.7	35.25	35.28	33.18	33.72	*10 dpi*	*Neg.*	*Neg.*	*Neg.*

*Neg.*: negative; highlighted in grey: sequenced samples; *: sequenced samples with low coverage; bold: negative whole genome influenza amplification; †: euthanized animal. Cells shaded in blue and green indicate vaccinated and nonvaccinated animals, respectively.

**Table 2:** Pathological results based on microscopic observation and quantitative scoring of HE and IHC staining lung samples, and percentage of lung-affected area.

Group	Pig ID	Euthanasia Day	Lung-Affected Area (%)	Histopathological Scoring	Immunohistochemistry for SIV
*Vaccinated Group*	*1*	*2 dpi*	3.28	*2*	++
	*2*		9.33	*3*	+++
	*3*		28.64	*3*	++
	*4*	*5 dpi*	13.22	*2*	-
	*5*		27.23	*3*	-
	*6*		7.23	*0.5*	-
	*7*	*10 dpi*	8.57	*1.5*	-
	*8*		9.76	*0.5*	-
		*mean*	13.41		
*Nonvaccinated Group*	*9*	*2 dpi*	29.52	*3*	+++
	*10*		33.65	*1.5*	+
	*11*		65.72	*3*	+
	*12*	*5 dpi*	24.5	*2*	+
	*13*		26.43	*3*	+
	*14*		35.06	*3*	+
	*15*	*10 dpi*	16.36	*1*	-
		*mean*	33.03		

Histopathology scoring: absence (0), few isolated (0.5), localized cluster (1), several (1.5–2), severely several (2.5), and many (3) airways affected. Moreover, minimal (1.5), mild (2) interstitial infiltrate, and plus moderate interstitial and alveolar infiltrates (2.5–3). SIV immunohistochemical scoring: absence (-), low (+), scattered (++), moderate (+++), and abundant (++++) amount of immunoreactivity.

## Data Availability

Sequencing data were deposited at NCBI, with the accession number (PRJNA763061).

## Supplementary Materials

The following are available online at https://www.mdpi.com/article/10.3390/v13102087/s1, Supplementary Table S1. Total number of reads obtained in each sequenced sample. Total number of reads and total number of reads mapped against reference genome after PCR duplicate removal in each influenza genome segment. Supplementary Table S2. SNVs and their effect on variants found in viral inoculum and samples collected from previously challenged vaccinated and nonvaccinated pigs with A/Swine/Spain/01/2010 (H1N1).

## References

[1] Swine Influenza: OIE—World Organisation for Animal Health. https://www.oie.int/en/animal-health-in-the-world/animal-diseases/Swine-influenza/.

[2] Das K., Aramini J.M., Ma L.-C., Krug R.M., Arnold E. (2010). Structures of influenza A proteins and insights into antiviral drug targets. Nat. Struct. Mol. Biol..

[3] Bouvier N.M., Palese P. (2008). The biology of influenza viruses. Vaccine.

[4] Lamb R.A., Lai C.J., Choppin P.W. (1981). Sequences of mRNAs derived from genome RNA segment 7 of influenza virus: Colinear and interrupted mRNAs code for overlapping proteins. Proc. Natl. Acad. Sci. USA.

[5] Lamb R.A., Choppin P.W., Chanock R.M., Lai C.J. (1980). Mapping of the two overlapping genes for polypeptides NS1 and NS2 on RNA segment 8 of influenza virus genome. Proc. Natl. Acad. Sci. USA.

[6] Chen W., Calvo P., Malide D., Gibbs J., Schubert U., Bacik I., Basta S., O’Neill R., Schickli J., Palese P. (2001). A novel influenza A virus mitochondrial protein that induces cell death. Nat. Med..

[7] Smith G.J.D., Vijaykrishna D., Bahl J., Lycett S.J., Worobey M., Pybus O.G., Ma S.K., Cheung C.L., Raghwani J., Bhatt S. (2009). Origins and evolutionary genomics of the 2009 swine-origin H1N1 influenza a epidemic. Nature.

[8] 2009 H1N1 Pandemic (H1N1pdm09 Virus) Pandemic Influenza (Flu) CDC. https://www.cdc.gov/flu/pandemic-resources/2009-h1n1-pandemic.html.

[9] Garten R.J., Davis C.T., Russell C.A., Shu B., Lindstrom S., Balish A., Sessions W.M., Xu X., Skepner E., Deyde V. (2009). Antigenic and genetic characteristics of swine-origin 2009 A(H1N1) influenza viruses circulating in humans. Science.

[10] Sun H., Xiao Y., Liu J., Wang D., Li F., Wang C., Li C., Zhu J., Song J., Sun H. (2020). Prevalent Eurasian avian-like H1N1 swine influenza virus with 2009 pandemic viral genes facilitating human infection. Proc. Natl. Acad. Sci. USA.

[11] Domingo E., Sheldon J., Perales C. (2012). Viral Quasispecies Evolution. Microbiol. Mol. Biol. Rev..

[12] Bergmann M., Garcia-Sastret A., Palese P. (1992). Transfection-Mediated Recombination of Influenza A Virus. J. Virol.

[13] Shao W., Li X., Goraya M.U., Wang S., Chen J.L. (2017). Evolution of influenza a virus by mutation and re-assortment. Int. J. Mol. Sci..

[14] Carrat F., Flahault A. (2007). Influenza vaccine: The challenge of antigenic drift. Vaccine.

[15] Nichol K.L., Treanor J.J. (2006). Vaccines for Seasonal and Pandemic Influenza. J. Infect. Dis..

[16] Ma W., Richt J.A. (2010). Swine influenza vaccines: Current status and future perspectives. Anim. Health Res. Rev..

[17] Reeth K.V., Brown I.H., Dürrwald R., Foni E., Labarque G., Lenihan P., Maldonado J., Markowska-Daniel I., Pensaert M., Pospisil Z. (2008). Seroprevalence of H1N1, H3N2 and H1N2 influenza viruses in pigs in seven European countries in 2002–2003. Influenza Other Res. Viruses.

[18] Simon G., Larsen L.E., Dürrwald R., Foni E., Harder T., Van Reeth K., Markowska-Daniel I., Reid S.M., Dan A., Maldonado J. (2014). European surveillance network for influenza in pigs: Surveillance programs, diagnostic tools and Swine influenza virus subtypes identified in 14 European countries from 2010 to 2013. PLoS ONE.

[19] Thacker E., Janke B. (2008). Swine Influenza Virus: Zoonotic Potential and Vaccination Strategies for the Control of Avian and Swine Influenzas. J. Infect. Dis..

[20] Reed L., Muench H. (1938). A simple method of estimating fifty per cent endpoints. Antioch Rev..

[21] Crisci E., Fraile L., Valentino S., Martínez-Guinó L., Bottazzi B., Mantovani A., Montoya M. (2014). Immune characterization of long pentraxin 3 in pigs infected with influenza virus. Vet. Microbiol..

[22] Bohorquez J.A., Muñoz-González S., Pérez-Simó M., Revilla C., Domínguez J., Ganges L. (2019). Identification of an Immunosuppressive Cell Population during Classical Swine Fever Virus Infection and Its Role in Viral Persistence in the Host. Viruses.

[23] Lopez E., Bosch-Camós L., Ramirez-Medina E., Vuono E., Navas M.J., Muñoz M., Accensi F., Zhang J., Alonso U., Argilaguet J. (2021). Deletion Mutants of the Attenuated Recombinant ASF Virus, BA71ΔCD2, Show Decreased Vaccine Efficacy. Viruses.

[24] López-Serrano S., Cordoba L., Pérez-Maillo M., Pleguezuelos P., Remarque E.J., Ebensen T., Guzmán C.A., Christensen D., Segalés J., Darji A. (2021). Immune Responses to Pandemic H1N1 Influenza Virus Infection in Pigs Vaccinated with a Conserved Hemagglutinin HA1 Peptide Adjuvanted with CAF^®^01 or CDA/αGalCerMPEG. Vaccines.

[25] Galindo-Cardiel I., Ballester M., Solanes D., Nofrarías M., López-Soria S., Argilaguet J.M., Lacasta A., Accensi F., Rodríguez F., Segalés J. (2013). Standardization of pathological investigations in the framework of experimental ASFV infections. Virus Res..

[26] Nielsen J., Bøtner A., Tingstedt J.E., Aasted B., Johnsen C.K., Riber U., Lind P. (2003). In utero infection with porcine reproductive and respiratory syndrome virus modulates leukocyte subpopulations in peripheral blood and bronchoalveolar fluid of surviving piglets. Vet. Immunol. Immunopathol..

[27] Sibila M., Aragón V., Fraile L., Segalés J. (2014). Comparison of four lung scoring systems for the assessment of the pathological outcomes derived from Actinobacillus pleuropneumoniae experimental infections. BMC Vet. Res..

[28] Busquets N., Segalés J., Córdoba L., Mussá T., Crisci E., Martín-Valls G.E., Simon-Grifé M., Pérez-Simó M., Pérez-Maíllo M., Núñez J.I. (2010). Experimental infection with H1N1 European swine influenza virus protects pigs from an infection with the 2009 pandemic H1N1 human influenza virus. Vet. Res..

[29] Sisteré-Oró M., López-Serrano S., Veljkovic V., Pina-Pedrero S., Vergara-Alert J., Córdoba L., Pérez-Maillo M., Pleguezuelos P., Vidal E., Segalés J. (2019). DNA vaccine based on conserved HA-peptides induces strong immune response and rapidly clears influenza virus infection from vaccinated pigs. PLoS ONE.

[30] Sabattini E., Bisgaard K., Ascani S., Poggi S., Piccioli M., Ceccarelli C., Pieri F., Fraternali-Orcioni G., Pileri S.A. (1998). The EnVision(TM)+ system: A new immunohistochemical method for diagnostics and research. Critical comparison with the APAAP, ChemMate(TM), CSA, LABC, and SABC techniques. J. Clin. Pathol..

[31] Detmer S.E., Gunvaldsen R.E., Harding J.C. (2013). Comparison of influenza a virus infection in high- and low-birth-weight pigs using morphometric analysis. Influenza Other Res. Viruses.

[32] Spackman E., Senne D.A., Myers T.J., Bulaga L.L., Garber L.P., Perdue M.L., Lohman K., Daum L.T., Suarez D.L. (2002). Development of a real-time reverse transcriptase PCR assay for type A influenza virus and the avian H5 and H7 hemagglutinin subtypes. J. Clin. Microbiol..

[33] Zhou B., Donnelly M.E., Scholes D.T., St. George K., Hatta M., Kawaoka Y., Wentworth D.E. (2009). Single-Reaction Genomic Amplification Accelerates Sequencing and Vaccine Production for Classical and Swine Origin Human Influenza A Viruses. J. Virol..

[34] Lycett S.J., Baillie G., Coulter E., Bhatt S., Kellam P., McCauley J.W., Wood J.L.N., Brown I.H., Pybus O.G., Brown Leigh A.J. (2012). Estimating reassortment rates in co-circulating Eurasian swine influenza viruses. J. Gen. Virol..

[35] Andrews S. FastQC: A Quality Control Tool for High Throughput Sequence Data. https://www.bioinformatics.babraham.ac.uk/projects/fastqc/.

[36] Bolger A.M., Lohse M., Usadel B. (2014). Trimmomatic: A flexible trimmer for Illumina sequence data. Bioinformatics.

[37] Langmead B., Salzberg S.L. (2012). Fast gapped-read alignment with Bowtie 2. Nat. Methods.

[38] Martín-Valls G.E., Simon-Grifé M., van Boheemen S., de Graaf M., Bestebroer T.M., Busquets N., Martín M., Casal J., Fouchier R.A.M., Mateu E. (2014). Phylogeny of Spanish swine influenza viruses isolated from respiratory disease outbreaks and evolution of swine influenza virus within an endemically infected farm. Vet. Microbiol..

[39] Danecek P., Bonfield J.K., Liddle J., Marshall J., Ohan V., Pollard M.O., Whitwham A., Keane T., McCarthy S.A., Davies R.M. (2021). Twelve years of SAMtools and BCFtools. Gigascience.

[40] Wickham H. (2016). Ggplot2: Elegant Graphics for Data Analysis.

[41] RStudio Open Source & Professional Software for Data Science Teams—RStudio. https://www.rstudio.com/.

[42] Wilm A., Aw P.P.K., Bertrand D., Yeo G.H.T., Ong S.H., Wong C.H., Khor C.C., Petric R., Hibberd M.L., Nagarajan N. (2012). LoFreq: A sequence-quality aware, ultra-sensitive variant caller for uncovering cell-population heterogeneity from high-throughput sequencing datasets. Nucleic Acids Res..

[43] Cingolani P., Platts A., Wang L.L., Coon M., Nguyen T., Wang L., Land S.J., Lu X., Ruden D.M. (2012). A program for annotating and predicting the effects of single nucleotide polymorphisms, SnpEff: SNPs in the genome of Drosophila melanogaster strain w1118; iso-2; iso-3. Fly.

[44] Nelson C.W., Moncla L.H., Hughes A.L. (2015). SNPGenie: Estimating evolutionary parameters to detect natural selection using pooled next-generation sequencing data. Bioinformatics.

[45] Nelson C.W., Hughes A.L. (2015). Within-host nucleotide diversity of virus populations: Insights from next-generation sequencing. Infect. Genet. Evol..

[46] VanderWaal K., Deen J. (2018). Global trends in infectious diseases of swine. Proc. Natl. Acad. Sci. USA.

[47] Ma W. (2020). *Swine* Influenza Virus: Current Status and Challenge. Virus Res..

[48] Wang R., Chen J., Gao K., Wei G.W. (2021). Vaccine-escape and fast-growing mutations in the United Kingdom, the United States, Singapore, Spain, India, and other COVID-19-devastated countries. Genomics.

[49] Wu N.C., Thompson A.J., Lee J.M., Su W., Arlian B.M., Xie J., Lerner R.A., Yen H.L., Bloom J.D., Wilson I.A. (2020). Different genetic barriers for resistance to HA stem antibodies in influenza H3 and H1 viruses. Science.

[50] Mancera Gracia J.C., Pearce D.S., Masic A., Balasch M. (2020). Influenza A Virus in Swine: Epidemiology, Challenges and Vaccination Strategies. Front. Vet. Sci..

[51] Sobel Leonard A., McClain M.T., Smith G.J.D., Wentworth D.E., Halpin R.A., Lin X., Ransier A., Stockwell T.B., Das S.R., Gilbert A.S. (2016). Deep Sequencing of Influenza A Virus from a Human Challenge Study Reveals a Selective Bottleneck and Only Limited Intrahost Genetic Diversification. J. Virol..

[52] Moncla L.H., Bedford T., Dussart P., Horm S.V., Rith S., Buchy P., Karlsson E.A., Li L., Liu Y., Zhu H. (2020). Quantifying within-host diversity of H5N1 influenza viruses in humans and poultry in Cambodia. PLOS Pathog..

[53] Fitch W.M., Leiter J.M., Li X.Q., Palese P. (1991). Positive Darwinian evolution in human influenza A viruses. Proc. Natl. Acad. Sci. USA.

[54] Li W., Shi W., Qiao H., Ho S.Y.W., Luo A., Zhang Y., Zhu C. (2011). Positive selection on hemagglutinin and neuraminidase genes of H1N1 influenza viruses. Virol. J..

[55] Machkovech H.M., Bedford T., Suchard M.A., Bloom J.D. (2015). Positive Selection in CD8+ T-Cell Epitopes of Influenza Virus Nucleoprotein Revealed by a Comparative Analysis of Human and Swine Viral Lineages. J. Virol..

[56] Pérez L.J., Díaz de Arce H., Perera C.L., Rosell R., Frías M.T., Percedo M.I., Tarradas J., Dominguez P., Núñez J.I., Ganges L. (2012). Positive selection pressure on the B/C domains of the E2-gene of classical swine fever virus in endemic areas under C-strain vaccination. Infect. Genet. Evol..

[57] de la Luna S., Fortes P., Beloso A., Ortín J. (1995). Influenza virus NS1 protein enhances the rate of translation initiation of viral mRNAs. J. Virol..

[58] Noah D.L., Twu K.Y., Krug R.M. (2003). Cellular antiviral responses against influenza A virus are countered at the posttranscriptional level by the viral NS1A protein via its binding to a cellular protein required for the 3′ end processing of cellular pre-mRNAS. Virology.

[59] White H.N. (2021). B-Cell Memory Responses to Variant Viral Antigens. Viruses.

[60] Fernandez-Sesma A., Marukian S., Ebersole B.J., Kaminski D., Park M.-S., Yuen T., Sealfon S.C., García-Sastre A., Moran T.M. (2006). Influenza virus evades innate and adaptive immunity via the NS1 protein. J. Virol..

[61] Carrillo B., Choi J.-M., Bornholdt Z.A., Sankaran B., Rice A.P., Prasad B.V.V. (2014). The Influenza A Virus Protein NS1 Displays Structural Polymorphism. J. Virol..

[62] Qian X.Y., Chien C.Y., Lu Y., Montelione G.T., Krug R.M. (1995). An amino-terminal polypeptide fragment of the influenza virus NS1 protein possesses specific RNA-binding activity and largely helical backbone structure. RNA.

[63] Li Y., Yamakita Y., Krug R.M. (1998). Regulation of a nuclear export signal by an adjacent inhibitory sequence: The effector domain of the influenza virus NS1 protein. Proc. Natl. Acad. Sci. USA.

[64] Ye Q., Krug R.M., Tao Y.J. (2006). The mechanism by which influenza A virus nucleoprotein forms oligomers and binds RNA. Nature.

[65] Xu R., Ekiert D.C., Krause J.C., Hai R., Crowe J.E., Wilson I.A. (2010). Structural basis of preexisting immunity to the 2009 H1N1 pandemic influenza virus. Science.

[66] Pang B., Cheung N.N., Zhang W., Dai J., Kao R.Y., Zhang H., Hao Q. (2016). Structural characterization of H1N1 nucleoprotein-nucleozin binding sites. Sci. Rep..

[67] Mitra S., Kumar D., Hu L., Sankaran B., Moosa M.M., Rice A.P., Ferreon J.C., Ferreon A.C.M., Prasad B.V.V. (2019). Influenza A Virus Protein NS1 Exhibits Strain-Independent Conformational Plasticity. J. Virol..

[68] Aragón T., de la Luna S., Novoa I., Carrasco L., Ortín J., Nieto A. (2000). Eukaryotic translation initiation factor 4GI is a cellular target for NS1 protein, a translational activator of influenza virus. Mol. Cell. Biol..

[69] Kochs G., García-Sastre A., Martínez-Sobrido L. (2007). Multiple Anti-Interferon Actions of the Influenza A Virus NS1 Protein. J. Virol..

[70] Zhou H.Z., Zhu J.Z., Tu J.T., Wei Z., Yong H., Zhengjun Y., Wensi Y., Yongtao L., Anding Z., Yurong W. (2010). Effect on Virulence and Pathogenicity of H5N1 Influenza A Virus through Truncations of NS1 eIF4GI Binding Domain. J. Infect. Dis..

[71] Gianfrani C., Oseroff C., Sidney J., Chesnut R.W., Sette A. (2000). Human memory CTL response specific for influenza A virus is broad and multispecific. Hum. Immunol..

[72] Nogales A., Chauché C., DeDiego M.L., Topham D.J., Parrish C.R., Murcia P.R., Martínez-Sobrido L. (2017). The K186E Amino Acid Substitution in the Canine Influenza Virus H3N8 NS1 Protein Restores Its Ability To Inhibit Host Gene Expression. J. Virol..

[73] Nemeroff M.E., Barabino S.M.L., Li Y., Keller W., Krug R.M. (1998). Influenza Virus NS1 Protein Interacts with the Cellular 30 kDa Subunit of CPSF and Inhibits 3′ End Formation of Cellular Pre-mRNAs. Mol. Cell.

[74] Chauché C., Nogales A., Zhu H., Goldfarb D., Ahmad Shanizza A.I., Gu Q., Parrish C.R., Martínez-Sobrido L., Marshall J.F., Murcia P.R. (2018). Mammalian Adaptation of an Avian Influenza A Virus Involves Stepwise Changes in NS1. J. Virol..

[75] Jiao P., Tian G., Li Y., Deng G., Jiang Y., Liu C., Liu W., Bu Z., Kawaoka Y., Chen H. (2008). A single-amino-acid substitution in the NS1 protein changes the pathogenicity of H5N1 avian influenza viruses in mice. J. Virol..

[76] Li J.J., Zhang K., Chen Q., Zhang X., Sun Y., Bi Y., Zhang S., Gu J., Li J.J., Liu D. (2018). Three amino acid substitutions in the NS1 protein change the virus replication of H5N1 influenza virus in human cells. Virology.

[77] Wang S., Zhang L., Zhang R., Chi X., Yang Z., Xie Y., Shu S., Liao Y., Chen J.-L. (2018). Identification of two residues within the NS1 of H7N9 influenza A virus that critically affect the protein stability and function. Vet. Res..

[78] Portela A., Digard P. (2002). The influenza virus nucleoprotein: A multifunctional RNA-binding protein pivotal to virus replication. J. Gen. Virol..

[79] Li Z., Watanabe T., Hatta M., Watanabe S., Nanbo A., Ozawa M., Kakugawa S., Shimojima M., Yamada S., Neumann G. (2009). Mutational Analysis of Conserved Amino Acids in the Influenza A Virus Nucleoprotein. J. Virol..

[80] Biswas S.K., Boutz P.L., Nayak D.P. (1998). Influenza Virus Nucleoprotein Interacts with Influenza Virus Polymerase Proteins. J. Virol..

[81] Pagani I., Di Pietro A., Oteiza A., Ghitti M., Mechti N., Naffakh N., Vicenzi E. (2018). Mutations Conferring Increased Sensitivity to Tripartite Motif 22 Restriction Accumulated Progressively in the Nucleoprotein of Seasonal Influenza A (H1N1) Viruses between 1918 and 2009. Msphere.

[82] Pietro A.D., Kajaste-Rudnitski A., Oteiza A., Nicora L., Towers G.J., Mechti N., Vicenzi E. (2013). TRIM22 Inhibits Influenza A Virus Infection by Targeting the Viral Nucleoprotein for Degradation. J. Virol..

[83] Russell C.J. (2021). Hemagglutinin Stability and Its Impact on Influenza A Virus Infectivity, Pathogenicity, and Transmissibility in Avians, Mice, Swine, Seals, Ferrets, and Humans. Viruses.

[84] Krammer F. (2019). The human antibody response to influenza A virus infection and vaccination. Nat. Rev. Immunol..

[85] Gamblin S.J., Haire L.F., Russell R.J., Stevens D.J., Xiao B., Ha Y., Vasisht N., Steinhauer D.A., Daniels R.S., Elliot A. (2004). The structure and receptor binding properties of the 1918 influenza hemagglutinin. Science.

[86] Murcia P.R., Hughes J., Battista P., Lloyd L., Baillie G.J., Ramirez-Gonzalez R.H., Ormond D., Oliver K., Elton D., Mumford J.A. (2012). Evolution of an Eurasian avian-like influenza virus in naïve and vaccinated pigs. PLoS Pathog..

[87] Kubiszewski-Jakubiak S., Worch R. (2020). Influenza A H1 and H3 Transmembrane Domains Interact Differently with Each Other and with Surrounding Membrane Lipids. Viruses.

[88] McAuley J.L., Gilbertson B.P., Trifkovic S., Brown L.E., McKimm-Breschkin J.L. (2019). Influenza Virus Neuraminidase Structure and Functions. Front. Microbiol..

[89] Gilchuk I.M., Bangaru S., Gilchuk P., Irving R.P., Kose N., Bombardi R.G., Thornburg N.J., Creech C.B., Edwards K.M., Li S. (2019). Influenza H7N9 Virus Neuraminidase-Specific Human Monoclonal Antibodies Inhibit Viral Egress and Protect from Lethal Influenza Infection in Mice. Cell Host Microbe.

[90] Östbye H., Gao J., Martinez M.R., Wang H., de Gier J.-W., Daniels R. (2020). N-Linked Glycan Sites on the Influenza A Virus Neuraminidase Head Domain Are Required for Efficient Viral Incorporation and Replication. J. Virol..

[91] Reeth K.V., Vincent A.L., Lager K.M. (2016). Vaccines and vaccination for swine influenza: Differing situations in Europe and the USA. Animal Influenza.

